# Reduced Contractility and Motility of Prostatic Cancer-Associated Fibroblasts after Inhibition of Heat Shock Protein 90

**DOI:** 10.3390/cancers8090077

**Published:** 2016-08-24

**Authors:** Alex Henke, Omar E. Franco, Grant D. Stewart, Antony C.P. Riddick, Elad Katz, Simon W. Hayward, Axel A. Thomson

**Affiliations:** 1 Medical Research Council, Centre for Reproductive Health, The Queens’s Medical Research Institute, Edinburgh EH16 4TJ, UK; dr.a.henke@gmail.com; 2 Department of Urologic Surgery and Cancer Biology, Vanderbilt University, Nashville, TN 37232-2765, USA; ofrancocoronel@northshore.org (O.E.F.); SHayward@northshore.org (S.W.H.); 3 Edinburgh Urological Cancer Group, Institute of Genetics and Molecular Medicine, University of Edinburgh, Edinburgh EH4 2XU, UK; grant.stewart@ed.ac.uk (G.D.S.); ariddick@doctors.org.uk (A.C.P.R.); 4 Division of Pathology, University of Edinburgh, Western General Hospital, Edinburgh EH4 2XU, UK; e.katz@dundee.ac.uk; 5 Division of Urology, Department of Surgery, Cancer Research Program, McGill University Health Centre Research Institute, 1001 Decarie Blvd, Montreal, QC H4A 3J1, Canada

**Keywords:** prostate cancer, cancer associated fibroblast, heat shock protein, contractility

## Abstract

*Background*: Prostate cancer-associated fibroblasts (CAF) can stimulate malignant progression and invasion of prostatic tumour cells via several mechanisms including those active in extracellular matrix; *Methods*: We isolated CAF from prostate cancer patients of Gleason Score 6–10 and confirmed their cancer-promoting activity using an in vivo tumour reconstitution assay comprised of CAF and BPH1 cells. We tested the effects of heat shock protein 90 (HSP90) inhibitors upon reconstituted tumour growth in vivo. Additionally, CAF contractility was measured in a 3D collagen contraction assay and migration was measured by scratch assay; *Results*: HSP90 inhibitors dipalmitoyl-radicicol and 17-dimethylaminoethylamino-17-demethoxygeldanamycin (17-DMAG) reduced tumour size and proliferation in CAF/BPH1 reconstituted tumours in vivo. We observed that the most contractile CAF were derived from patients with lower Gleason Score and of younger age compared with the least contractile CAF. HSP90 inhibitors radicicol and 17-DMAG inhibited contractility and reduced the migration of CAF in scratch assays. Intracellular levels of HSP70 and HSP90 were upregulated upon treatment with HSP90 inhibitors. Inhibition of HSP90 also led to a specific increase in transforming growth factor beta 2 (TGFβ2) levels in CAF; *Conclusions*: We suggest that HSP90 inhibitors act not only upon tumour cells, but also on CAF in the tumour microenvironment.

## 1. Introduction

Carcinomas are complex multi-cellular communities, consisting of tumour epithelia, immune cells, stroma, and the surrounding microenvironment. The relationships between these tissues evolves as the tumour progresses from a localized well differentiated lesion to an invasive and less organized structure. In recent years the tumour microenvironment (TME) has become increasingly recognized as an active and functionally important regulator of tumour progression and response to treatment. The TME includes many cell types including fibroblasts, nerves, endothelial cells and pericytes and immune/inflammatory cells, as well as components of the organ-specific stroma such as smooth muscle and adipocytes. Cancer-associated fibroblasts (CAF) were identified as active, pro-tumourigenic key players in the stroma, as they can promote malignant progression in initiated but non-tumourigenic epithelial cells [1]. CAF communicate with, and regulate the proliferation, of adjacent epithelia via multiple signalling molecules, e.g., CXCL12 and TGFβ [2,3,4]. They can also modulate the local extracellular matrix by secretion of matrix metalloproteinases (MMP) and deposition of hyaluron and collagen [5], thereby stiffening the tissue. The increased tissue stiffness in itself may act as a tumour-promoting, physical cue during tumour progression [6]. CAF can create paths for tissue invasion of epithelial cells [7,8], which requires contractility and motility [7,8,9]. Inhibition of CAF contractility or motility is a potential therapeutic target.

The TME and CAF are targets for a variety of approaches to inhibit tumour growth, though at present few are sufficiently potent to be primary treatments [10]. Cancer cells show considerable genetic instability, allowing them to evolve mechanisms to escape therapy leading to eventual disease relapse. In contrast, cells of the TME appear to be genetically stable [11] although there may be epigenetic changes in CAF. Hence, drugs targeting the TME may be less subject to development of resistance compared to tumour cell targeted therapies. Drugs targeting TME could be indirect anti-tumour agents that might work in conjunction with conventional agents, a recognition that has already led to some development and clinical testing [12].

HSP90 is a highly abundant and ubiquitously expressed protein with involvement in many cellular processes. Although HSP90 is not a conventional target for cancer therapy, several inhibitors have been developed some of which are being tested in clinical trials [13,14]. HSP90 inhibitors may target directly the tumour cells to inhibit motility and invasion [15], or by indirect effects via angiogenesis [16,17]. There is also evidence for effects of HSP90 inhibitors upon fibroblast migration and fibrosis [18,19] as well as direct inhibition of CAF tumour interactions [20]. Other studies demonstrated secretion of extracellular HSP90 by prostate tumour cells to stimulate the conversion of fibroblasts into CAF [21,22]. These studies illustrate that HSP90 inhibition may have effects upon several aspects of tumour growth; both direct as well as indirect.

In this study we tested the HSP90 antagonists radicicol and 17-DMAG upon CAF-induced tumour growth, and observed inhibition of tumour size and proliferation. We used a 3D collagen contraction assay to examine CAF from patients of different ages and pathological features and tested the effects of HSP90 inhibitors in this assay. HSP90 inhibition led to a reduction in collagen contractility and reduced CAF migration. We suggest that some effects of HSP90 inhibition upon tumour growth in vivo may be mediated via CAF, in addition to direct effects previously documented upon tumour cells.

## 2. Results

### 2.1. CAF Isolation

**Figure 1:**
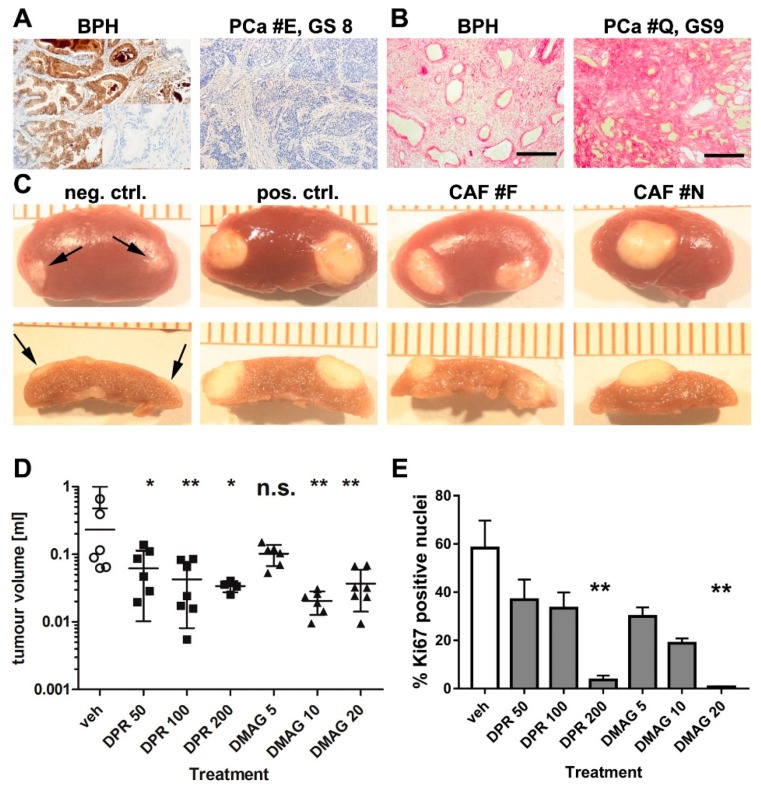
Characterisation of cancer-associated fibroblasts (CAF) and inhibition of tumour size and proliferation after treatment with HSP90 inhibitors. (**A**) Immunohistochemistry for Microseminoprotein (MSMB) showed positive staining in non-malignant benign prostatic hyperplasia BPH tissue (left panel; inset: antibody control) but was mostly absent in PCa (right panel) and was used to confirm tumour presence in tissue from which CAF were derived. Microscopy with 10× objective lense; PCa panel has identical scale as images in (B) (scale bar 200 µM). BPH images also with 10× objective lense but illustration at half the size as the other images in (A,B); (**B**) Collagen was stained by picrosirius red in BPH (non-malignant) and prostate cancer with a Gleason score of 9. Collagen was more abundant in prostate cancer samples. Microscopy with 10× objective; scale bar 200 µM; (**C**) Recombination of normal prostate fibroblasts (neg. ctrl., negative control) with BPH1 cells and kidney capsule grafting produced little growth (arrows). Recombination of previously validated CAF (pos. ctrl., positive control) and CAF isolates F and N with BPH1 cells led to tumour growth, demonstrating pro-tumourigenic activity of our primary CAF. Images in the upper row shows gross morphology, while the lower row shows cross sections of tumours used for size measurements; (**D**) CAF/BPH1 tumours were grown for 2 months followed by a 1 month treatment with vehicle control (veh), dipalmitoyl-radicicol (DPR) at 50, 100 and 200 mg/kg or with 17-DMAG (DMAG) at 5, 10 and 20 mg/kg. Tumour size was reduced after treatment with dipalmitoyl-radicicol at all doses, and with DMAG at 10 or 20 mg/kg. The vehicle control group was the same for both treatment groups since tests were performed simultaneous in parallel. Data are presented as mean + S.E.M. and asterisks denote the level of significance * for *p* < 0.05, ** for *p* < 0.01 and *** for *p* < 0.001. n.s. = not significant. Statistical test: one-way ANOVA. Individual data are provided in Supplementary Table S2; (**E**) To examine effects upon cell proliferation within tumours, tissue sections of CAF/BPH1 tumours were stained for the proliferation marker Ki67, and positive nuclei counted. The highest doses of DPR treatment (200 mg/kg) and DMAG treatment (20 mg/kg) resulted in a significantly decreased Ki67 index. n = 3; one way-ANOVA, data are presented as mean + S.E.M. and asterisks denote the level of significance as mentioned above.

Tissue from patients undergoing transurethral resection of the prostate (TURP) with a prior cancer diagnosis were used for CAF isolation. We isolated prostatic CAF from TURP samples and grew 20 PCa CAF cultures from 40 patients with a Gleason Score of six or higher, or poorly differentiated carcinoma. Routine pathology on biopsies before surgery and on TURP samples after surgery confirmed the presence of PCa. Supplementary Table S1 gives an overview of patients from whom CAF were derived. Microseminoprotein (MSMB) beta is a protein expressed in normal and BPH prostate but is decreased or absent in PCa [23], and we stained patient samples for MSMB to confirm the presence of tumour in the tissue specimens. While BPH control tissue showed strong MSMB staining, this was low or absent in PCa specimens, as shown in Figure 1A.

Cellular heterogeneity of CAF populations is well documented in vivo and in vitro [4,24,25,26]. Some organs, such as breast, contain few myofibroblasts, and hence an increase of the proteins alpha smooth actin (αSMA) and vimentin (VIM) is considered to be a marker of CAF in these tissues. In the prostate, smooth muscle cells are abundant and therefore αSMA is not a reliable CAF marker [27]. However, reactive stroma can be identified by picrosirius red staining [28,29,30], which detects collagen deposition. Picrosirius red staining of our samples of BPH and PCa samples are shown in Figure 1B, and PCa tissue showed extensive collagen-positive areas while these were absent or less extensive in BPH tissue. A previous publication from our laboratory demonstrated expression of VIM, SMACT (Smooth Muscle Alpha Acti), FSP (Fibroblast Specific Protein) and smooth muscle marker CNN (Calponin) in CAFs [24].

We used an in vivo xenograft assay to demonstrate tumour-promoting properties of our CAF. CAF from 11 patients were recombined with non-tumourigenic but initiated prostatic epithelial cells (BPH1), encased in a collagen matrix, and grafted under the kidney capsule of immune-deficient SCID mice. After three months, kidneys and grafts were explanted and tumour size measured, and volume estimated using an ellipsoid formula [1]. All CAF populations initiated tumour growth in BPH1 cells. Control normal primary fibroblasts (Figure 1C), which were obtained from a histological normal region from a patient with prostate cancer, who underwent radical prostatectomy, did not form tumours. Hence, in this bioassay we demonstrated that the fibroblast populations showed pro-tumourigenic CAF-activity, consistent with previously published studies [1].

### 2.2. The Effects of HSP90 Inhibitors upon CAF-Induced Tumourigenesis in Vivo

Our interest in HSP90 emerged from studies where we used small molecule inhibitors of signalling pathways in vitro, and observed a significant effect of inhibitors with documented “off target” effects upon HSP signaling. This led us to try HSP90 inhibitors directly. We studied the effects of HSP90 inhibitors upon tumours reconstituted from CAF and BPH1 cells which were allowed to develop for 2 months prior to the start of treatment with HSP90 inhibitors. This is a translationally relevant model of patient tumours that might undergo treatment with HSP90 inhibitors. We chose to assay the effects of 14,16-dipalmitoyl-radicicol and 17-DMAG which are structurally independent HSP90 inhibitors. Radicicol was reported to be ineffective in vivo, but a lipidated derivative, 14,16-dipalmitoylradicicol, showed anti-tumour activity in vitro and in vivo [31]. In order to exclude possible off-target effects and confirm the findings with dipalmitoyl-radicicol, we also used 17-dimethylaminoethylamino-17-demethoxygeldanamycin (17-DMAG), an HSP90 inhibitor that is structurally unrelated to radicicol. At the time when this study was initiated, other HSP90 inhibitors such as AUY922 or ganetisib were not available and these newer inhibitors show better efficacy. Two CAF populations were used to generate CAF/BPH1 recombinants and xenografted into SCID mice with three grafts per kidney. The tumours were grown for two months before the start of i.p. injections every four days over one month with 0, 50, 100 and 200 mg/kg dipalmitoyl-radicicol or 0, 5, 10 and 20 mg/kg 17-DMAG. Despite sample heterogeneity, the HSP90 inhibitor-treated animals had significantly lower tumour volumes than the vehicle control-treated animals (Figure 1D). One animal in the group receiving the highest dose of dipalmitoyl-radicicol died due to unknown causes. HSP90 inhibitors have been shown to cause liver toxicity in an animal model of gastrointestinal cancer [32] and also in patients with castration-resistant prostate cancer in a phase II clinical trial for a novel HSP90 inhibitor [33]. Nevertheless, the reduction in tumour size using dipalmitoyl-radicicol was statistically significant at 100 mg/kg, while 17-DMAG at either 10 or 20 mg/kg elicited a significant reduction in tumour size.

Next, we examined effects of treatment with HSP90 inhibitors upon cellular proliferation using nuclear Ki67 expression in tissue sections of xenografts after treatment. Histology of the tumours is shown in Supplementary Figure S1. We observed a dose-dependent reduction in Ki67 staining after treatment of tumours with dipalmitoyl-radicicol and 17-DMAG (Figure 1E). Quantitative analysis demonstrated a substantial reduction in Ki67-positive nuclei from 58% in the control group to 3.6% in the highest dose dipalmitoyl-radicicol treatment group and 0.8% in the 17-DMAG group (*p* = 0.0079 and *p* = 0.0010, respectively; one-way ANOVA) (Figure 1D). Taken together, the effects upon tumour size and cellular proliferation indicated that inhibition of HSP90 reduced tumour cell growth, albeit with a potential narrow therapeutic dosage window. 17-DMAG appeared to be better tolerated than di-palmitoyl-radicicol in vivo.

### 2.3. Effects of HSP90 Inhibitors Upon CAF Contractility in Vitro

**Figure 2:**
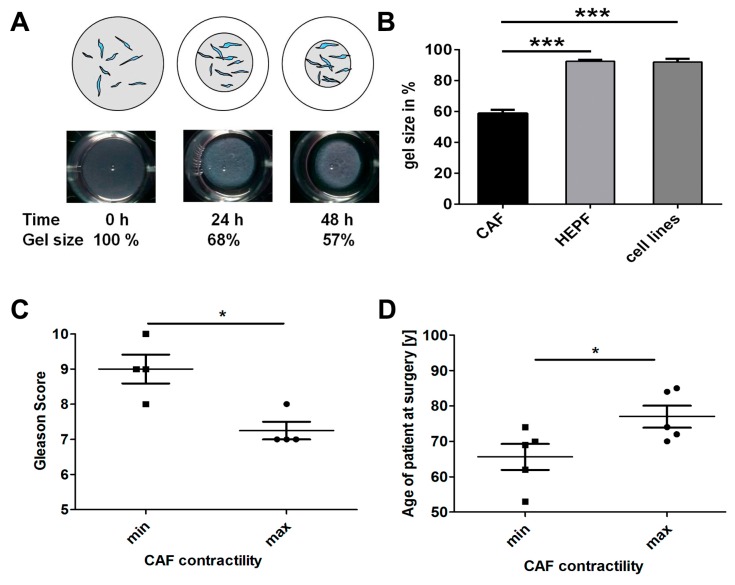
Contractility of CAF in vitro and comparison with Gleason grade and age. (**A**) Top: schematic diagram illustrating the collagen gel contraction (CGC) assay. Fibroblasts were embedded into a collagen matrix (grey) which contracted during culture period followed by measurement and calculation of percentage contraction; Bottom: images of a collagen gel at 0, 24 and 48 h showing a 68%–57% reduction in size; this corresponds to a contractility of 0%, 32% and 43%; (**B**) Comparison of collagen contractility of CAF with human embryonic fibroblasts and fibroblast cell lines after 48 h. CAF (*n* = 20 different primary lines) contracted collagen gels while human embryonic prostatic fibroblasts (HEPF) (*n* = 4 different primary lines) and fibroblast cell lines (NIH3T3 & WPMY-1; *n* = 2) showed significantly less contractility (*p* < 0.0001 and *p* = 0.0002, respectively; unpaired, two-tailed *t*-test); (**C**) Comparison of contractility with Gleason score, when comparing most and least contractile quartiles. The highest contractility was demonstrated by CAF lines from patients with a significantly lower Gleason score than in CAF lines from patients with the lowest contractility rate and highest Gleason score (48 h of incubation, *n* = 4 per group, two-tailed Mann Whitney test, *p* = 0.036); (**D**) The age of donors was significantly lower in the most versus the least contractile CAF lines (48 h of incubation, *n* = 5 per group; unpaired, two-tailed *t*-test, *p* = 0.046). (B–D) Data are presented as mean + S.E.M. and asterisks denote the level of significance: * for *p* < 0.05, ** for *p* < 0.01 and *** for *p* < 0.001.

We examined the ability of CAF to contract collagen gels in a 3D assay, and examined patient characteristics as well as effects of HSP90 inhibitors. We modified the assay to improve reproducibility by complete dislodgement of gels from bottom and walls of the wells and brief temporary removal of medium for imaging and subsequent quantitation. Figure 2A shows contraction of a collagen gel over 24 and 48 h. Contractility in a 3D collagen lattice was observed for CAF, but not for embryonic prostate fibroblasts or fibroblast cell lines (Figure 2B). Prostatic CAF reduced the gel area to an average of 59% (SD = 10.3%) of the original size after 48 h (*n* = 20 CAF isolates), while primary human embryonic prostatic fibroblasts (HEPF) had only minimal contractility (*n* = 4 samples). Also the NIH3T3 fibroblast cell line and the human prostatic myofibroblast cell line WPMY-1 (Figure 2B) showed little contractility. We demonstrated that CAF had a significantly enhanced contractility compared to other fibroblasts (CAF vs. HEPF *p* < 0.0001, and CAF vs. cell lines *p* = 0.0002).

### 2.4. CAF Contractility and Patient Parameters

We examined potential correlations between patient clinical parameters and CAF contractility. We observed no significant correlation of contractility of our CAF (*n* = 20 lines) with Gleason Score or age, possibly due to low sample number, though there appeared to be a modest trend (Supplementary Figure S2). Thus, we re-examined the extreme cases of contractility of the upper and lower quartiles. Two CAF isolates were from poorly differentiated PCa, and since the patients were receiving hormone treatment they were not assigned a Gleason score. This reduced the number of CAF to four for each contractility group of the most versus the least contractile CAF. The least contractile CAF were derived from patients with significantly higher Gleason Score than the most contractile CAF isolated from patients with lower Gleason scores (Figure 2C). When the three most and three least contractile CAF lines were xenografted with BPH-1 cells into mice, the highly contractile CAF produced larger tumours than the less contractile CAF but this difference was not statistically significant, possibly due to high intra-group variability (Supplementary Figure S3). When age and CAF contractility were compared, CAF from younger patients exhibited lower contractility than older patients (Figure 2D).

### 2.5. Analysis of HSP Inhibitors upon Contracility and Potential Cytotoxicity

**Figure 3:**
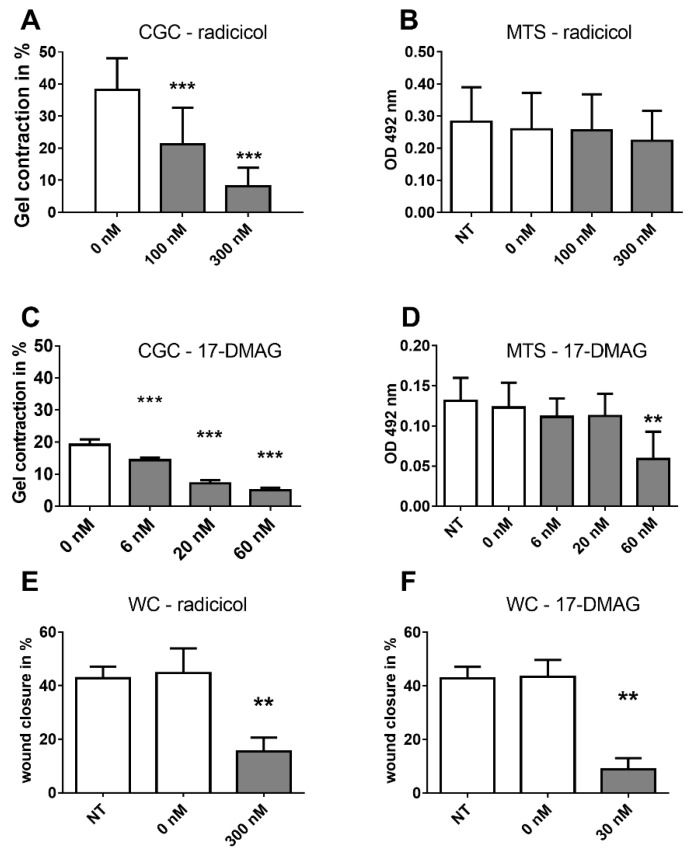
Effects of HSP90 inhibitors upon CAF contractility and mobility. (**A**) Radicicol inhibited collagen gel contraction (CGC) by CAF in a dose-dependent manner (*p* < 0.001 for all doses and time points vs. vehicle control; *n* = 21 experiments in triplicates for 0 nM, *n* = 18 for 100 nM and *n* = 16 for 300 nM with a total of 11 different CAF lines; unpaired two-sided *t*-test); (**B**) Treatment with radicicol did not reduce cell viability, as measured with an MTS assay after the CGC (*n* = 10 independent experiments and CAF lines; repeated measures ANOVA, *p* > 0.05; not significant). NT = no treatment; 0 nM received the dose of vehicle comparable to the highest treatment dose; (**C**) 17-DMAG significantly inhibited collagen gel contraction in a dose-dependent manner (*n* = 3 CAF lines and independent experiments in triplicates; repeated measures ANOVA, *p* < 0.001 for all doses); (**D**) 17-DMAG showed little effect upon cell viability in an MTS assay of CAF after the CGC except for the highest dose of 60 nM (*n* = 3 independent experiments and CAF lines; repeated measures ANOVA, *p* < 0.01); (**E**) Radicicol treatment (300 nM) reduced CAF migration in wound closure (WC) in the scratch assay after 24 h. *n* = 5 CAF populations, paired, two-sided *t*-test. NT = no treatment; (**F**) 30 nM 17-DMAG reduced migration in the scratch assay in CAF after 24 h. *n* = 5 CAF populations, *p* < 0.01 paired, two-sided *t*-test. All panels: mean + S.E.M. and asterisks denote the level of significance: * for *p* < 0.05, ** for *p* < 0.01 and *** for *p* < 0.001. Incubation times: CGC 48 h, MTS 3 h, WC 24 h.

We determined the effects of radicicol and 17-DMAG upon CAF contractility in the 3D collagen gel contraction (CGC) assay. To ensure that effects were not due to cellular toxicity of the HSP90 inhibitors, we used a sensitive colorimetric assay of viable cells within the collagen gels, based on the NAD(P)H-dependent reaction of dehydrogenase enzymes that converts a tetrazolium salt (MTS) into formazan. We identified radicicol as a potent inhibitor of CAF contractility in the 200–300 nanomolar range (Figure 3A). We observed little or no cytotoxicity of radicicol in the MTS assay (Figure 3B). This suggests that radicicol treatment had a direct effect upon contractility rather than indirect effects via cell toxicity. We repeated our analysis with 17-DMAG, which significantly impeded CAF contractility without cytotoxic effects in a dose-responsive fashion at nanomolar concentrations (Figure 3C,D), though the highest dose led to some cell toxicity. In addition, we used dorsomorphin (also known as compound C), which showed significant inhibition of contractility (Supplementary Figure S4). Dorsomorphin has been reported to inhibit both BMP signalling as well as HSP90 and other off-target effects [34]. We suggest that the effect upon contractility was via HSP90 effects rather than via BMP inhibition, as the use of another, specific BMP inhibitor (LDN-193189) did not affect contractility in our experiments. Taken together, the inhibitors targeting HSP90 showed significant effects upon CAF contractility.

### 2.6. Effects of HSP90 Inhibitors upon Motility

The effect of HSP90 inhibitors was also tested in a wound closure (WC, scratch) assay to address effects upon CAF motility. The open area of scratches in vitro was quantified with ImageJ directly after scratch initiation (t = 0 h) and after 24 h of culture with or without HSP90 inhibitors. Both, radicicol and 17-DMAG, significantly reduced the migration of CAF into the scratch area in comparison with controls (*p* = 0.0048 and 0.0036, respectively) (Figure 3E,F). We conclude that HSP90 inhibition significantly impaired the motility of CAF.

### 2.7. HSP90 Inhibitors and Secretion of TGFβ

**Figure 4:**
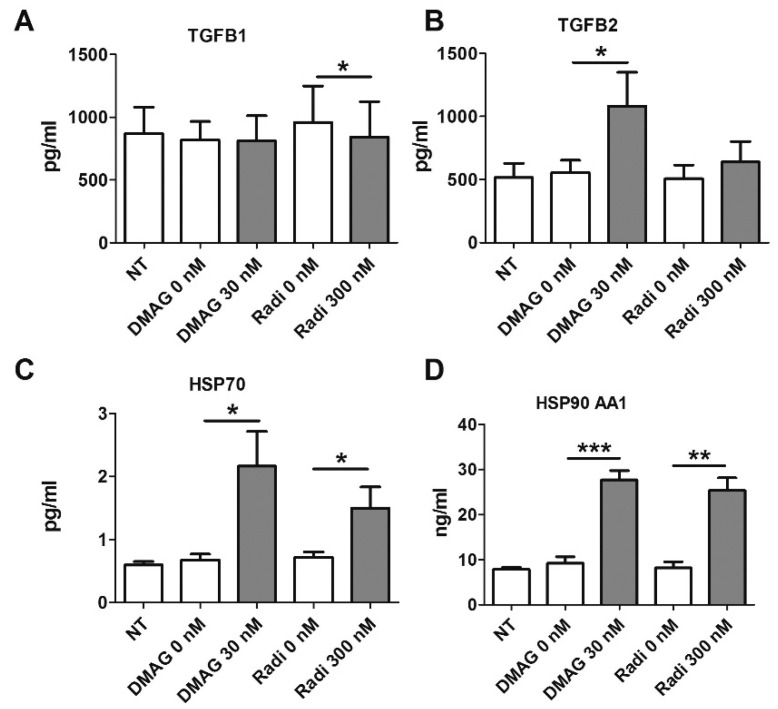
Multiplex bead assay to measure TGFβ and HSP protein levels in CAF following HSP inhibitor treatment. CAF were treated with Radicicol or 17-DMAG and conditioned medium was assayed for secreted TGFβ1 (**A**) and TGFβ2 (**B**); TGFβ1 levels remained the same or were slightly decreased by radicicol treatment, while TGFβ2 secretion increased upon treatment with 17-DMAG. Intracellular levels of HSP70 (**C**) and HSP90 AA1 (**D**) were measured in CAF cell lysates. Increased levels of both HSP70 and HSP90 AA1 were observed after treatment with both, 17-DMAG and radicicol (*n* = 4 different CAF lines measured in duplicates; paired, one-sided *t*-tests). All panels: mean + S.E.M. and asterisks denote the level of significance as mentioned above.

CAF overexpress many chemokines and growth factors including transforming growth factor beta (TGFβ). TGFβ has cell compartment-specific autocrine and paracrine effects, it contributes to tumour cell proliferation by up-regulating CXCR4 in epithelial cells, and enhancing invasion [3,35]. It also regulates fibroblast and smooth muscle cell differentiation [27,36,37], which may be relevant to contractility by altering CAF differentiation and subsequent indirect effects upon contractility. The secretion of TGFβ by CAF was measured in conditioned medium from CAF exposed to HSP90 inhibitors or vehicle control for 24 h, using a multiplex bead assay. While 17-DMAG had no effect, Radicicol led to a very small but significant down-regulation of TGFβ1 protein (Figure 4A). In contrast, the secretion of TGFβ2 protein was significantly up-regulated by 17-DMAG but not by radicicol (Figure 4B). The levels of TGFβ3 were below detection limits in all cases. In summary, there was a compound-dependent and mixed effect on TGFβ protein secretion by CAF exposed to HSP90 inhibitors. To determine whether the increase in TGFβ secretion might be mediating some effects upon contractility, we added recombinant human TGFβ2 protein and TGFβ-inhibiting antibodies to CAF in the CGC, however, these had no effect on contractility.

### 2.8. HSP Levels Following Treatment with HSP90 Inhibitors

Next, we examined intracellular protein levels of heat shock proteins in CAF after treatment with HSP90 inhibitors. Lysates of treated CAF were subjected to a multiplex bead assay for HSP70, HSP90 AA1, HSP27 and HSP60. Both 17-DMAG and radicicol led to a significant increase of HSP70 (Figure 4C) and HSP90 isoform AA1 in the cytosol of lysed CAF (Figure 4D). Radicicol had no significant impact on total or phosphorylated HSP27 or HSP60, but 17-DMAG treatment resulted in a decrease in phosphorylated HSP27 and an increase in HSP60 (Supplementary Figure S5). These data confirm that HSP90 inhibitors lead to increased intracellular levels of HSP70 and HSP90. Overexpression of HSPs after treatment with small molecules inhibitors has been observed in other systems, consistent with our observations [15,17], and suggest that our inhibitors were eliciting effects in CAF.

### 2.9. Effects of HSP90 Inhibitors upon ROCK in Vitro

**Figure 5:**
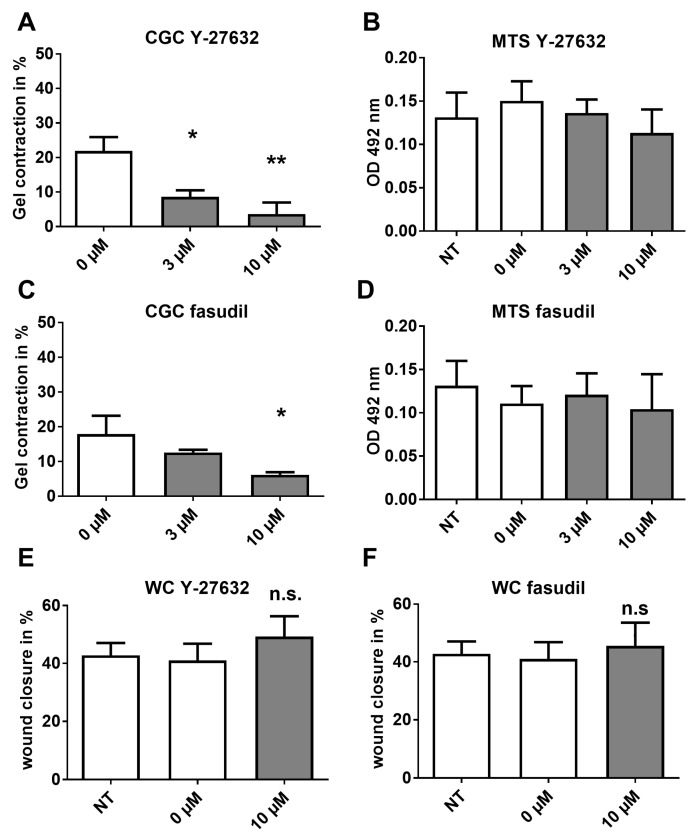
Contractility but not motility regulated via the ROCK pathway. Effects of ROCK inhibitors upon CAF collagen contractility was measured, after which the cell viability was examined by MTS assay. (**A**) The ROCK inhibitor Y-27632 impaired CAF contractility and showed no cytotoxic effects in an MTS assay (**B**); (**C**) The ROCK inhibitor fasudil inhibited CAF contractility with little cytotoxicity (**D**); In the scratch assay, Y-27632 (**E**) and fasudil (**F**) had no effect. (**A**–**D**) CAF from *n* = 3 different patients; repeated measures ANOVA; (**E**,**F**) CAF from *n* = 4 patients; paired, two-sided *t*-test. All panels: mean + S.E.M. and asterisks denote the level of significance * for *p* < 0.05, ** for *p* < 0.01 and *** for *p* < 0.001. n.s. = not significant. Incubation times: CGC 48 h, MTS 3 h, WC 24 h.

Recent studies have shown that CAF can create paths through the ECM (Extra Cellular Matrix) which tumour cells follow during invasion, and this process is dependent on CAF cellular contractility controlled by the Rho/ROCK pathway [8]. Therefore, we examined whether ROCK (Rho-associated protein kinase) inhibition via two structurally different inhibitors was also able to impair CAF contraction of gels directly, without cytotoxic effects. Y-27632 significantly decreased collagen gel contraction (*n* = 3 CAF lines; *p* = 0.0041; 1 way ANOVA) (Figure 5A) without cytotoxic effects as measured via the MTS assay, despite of high doses of 10 µM (Figure 5B). A second, structurally unrelated compound, fasudil, also significantly reduced collagen gel contraction (*n* = 3 CAF lines; *p* = 0.0256; 1 way ANOVA) without cellular cytotoxicity (Figure 5C,D). However, when the inhibitors were used in the scratch assay, we found no inhibition of the motility of CAF (Figure 5E,F), in contrast to observations using the HSP90 inhibitors.

## 3. Discussion

In the current study, we used primary prostate cancer CAF cultures and showed functional impairment in contractility and motility following HSP90 inhibition. Furthermore, reconstituted CAF/tumour cell xenografts were smaller after treatment with HSP90 inhibitors in vivo, which may be relevant to patient treatment given the translational model system used. Another study has recently identified a novel HSP90 inhibitor of CAF activity in an oral squamous carcinoma model [38], which is consistent with the data presented here. Together, these studies suggest that inhibition of HSP90 action in CAF can reduce tumour growth, and that these effects are likely in addition to direct effects upon tumour cells themselves. Additionally, a recently published study suggested that inhibition of extracellular HSP90alpha derived from prostate tumour cells can convert normal prostatic fibroblasts into CAF [22]. Thus, HSP90 inhibitors could interfere with pro-tumourigenic activities in tumour cells themselves, their secreted extracellular HSP90, as well as via intracellular HSP90 in CAF.

Most studies of HSP90 inhibition have focussed upon direct effects in tumour cell lines without consideration of CAF or TME effects [37,39]. Our approach augments these studies as we have studied HSP90 effects on CAF in vitro and reconstituted CAF-mediated tumours in vivo. We observed that CAF-dependent tumour growth of BPH1 cells was impeded by HSP90 inhibitors in vivo. Taken together, these studies imply that HSP90 inhibition not only affects prostate tumour cells directly but also CAF and the interactions between these cell types.

Studies on the presence of reactive stroma in human prostate tumours have demonstrated that stromal histology can predict patient outcome [30]. Heterogeneity within the TME and CAF populations is well documented and there may be co-evolution between CAF or CAF subsets within patient subgroups [40]. Therefore, we speculated that CAF properties may change during disease progression, and examined whether there were changes in contractility in CAF isolated from tumours with low or high Gleason score. There was no overall correlation between Gleason score and contractility, but a selective analysis of the most and least contractile CAF did show an inverse correlation with Gleason score. Similarly, we observed a correlation between patient age and CAF contractility such that the most contractile CAF were found in younger patients and the least contractile CAF were found in older patients. When our most and least contractile CAF were assayed in tumour reconstitution with BPH1 cells in vivo, there was a suggestion that the most contractile CAF led to greater tumour size, however this was not statistically significant. We conclude that further investigation with larger numbers of CAF is required to determine if there is a correlation between contractility and pro-tumourigenic activity of CAF.

Treatment of CAF with HSP90 inhibitors is likely to affect several parameters of cell homeostasis as well as secretion of chemokines and growth factors. We chose to examine secreted TGFβ, for which both, pro- and anti-tumourigenic effects have been described in the literature [41,42]. HSP90 inhibition with 17-DMAG surprisingly increased TGFβ2 secretion by CAF, with no effect upon TGFβ1 or TGFβ3. The increased secretion of TGFβ2 may be augmented by stroma-specific expression of cathepsin D, as secreted TGFβs are bound to the ECM in a latent, inactivated form [43]. It is conceivable that increased levels of proteases lead to a higher TGFβ2 release via cleavage from extracellular sources or that there is a direct effect upon TGFβ2 synthesis and secretion. This increase in TGFβ2 may exert several effects upon tumour growth in vivo, however, we saw no direct effects of TGFβ2 or TGFβ2 inhibition upon CAF contractility. The physiological effect of elevated TGFB2 on the tumour could potentially result in increased extravasation [41] but which is reduced following HSP90 inhibition.

HSP70 and HSP90 proteins were found to be up-regulated after exposure to HSP90 inhibitors. Heat shock factor protein 1 (HSF1) is an HSP90 client and HSP90 inhibition leads to HSF1 dissociation from HSP90 and subsequent HSF1 activation, which in turn is a transcription factor for HSP90. A recent study suggested dual inhibition of HSF1 and HSP90 for improved tumour reduction [44].

Regarding their mechanism of action, HSP90 inhibitors have pleiotropic effects. In respect to contractility, we suggest that the Rho/ROCK pathway may be active since ROCK inhibitors mimicked the effect of HSP90 inhibitors in the contraction assay. Motility was affected by HSP90 but not by ROCK inhibitors, suggesting that additional effector pathways were inhibited by radicicol and 17-DMAG. This view is in accordance with a recent study in which HSP90 interaction with its human client proteins was quantified. HSP90 bound 7% of the transcription factors, but 30% of ubiquitin ligases and 60% of kinases investigated [45]. Interestingly, both ROCK and one of its downstream effectors, focal adhesion kinase (FAK), were reported to be weak interaction partners of HSP90 in this study. This might explain why the HSP90 inhibitors showed effects upon motility, but the ROCK inhibitors did not.

Since hundreds of HSP90 clients have been confirmed and identified, it is likely that HSP90 inhibitory effects on CAF and other cells results from combinatorial inhibitory effects on several different pathways. Novel and potentially interesting HSP90 inhibitors have been tested in preclinical cancer studies with focus on the tumour cells but most have not entered clinical trials for prostate cancer yet [38,46,47,48,49,50]. There are clinical trials with results for phase I and II, while phase III trials are ongoing. Compound-specific efficacies and side effects were identified. The compound retaspimycin hydrochloride (IPI-504) was tested in a phase II trial on 19 patients with castration-resistant prostate cancer (CRPC) with little or no efficacy but severe side effects [33]. In contrast, for a phase I clinical trial for 17-DMAG, over 20 patients were enrolled, who suffered different types of solid malignancies, including CRPC, which were partially responsive to treatment. This was a dose escalation study, and doses of <80 mg/m^2^ were well tolerated with tolerable adverse events. Unfortunately, the maximal dose of 106 mg/m^2^ resulted in severe side effects, including death [51].

This suggests that HSP90 inhibitors may be of use in prostate cancer and other malignancies. There is no HSP90 inhibitor that has successfully completed a phase III trial yet or gained market approval, as the compounds were either difficult to synthesise, structurally unstable or too toxic. However, these candidates were based on geldanamycin as original lead compounds and constitute the so-called first generation of HSP90 inhibitors. The 2nd generation of HSP90 inhibitors appear to have less toxicity and are currently under development in clinical trials phase I to III, e.g., ganetispib (Synta) or AUY922 (Novartis) (reviewed in [14,52,53] and references therein).

In summary, our study utilised primary prostate cancer CAF and demonstrated that key characteristics such as contractility and motility can be inhibited by HSP90 inhibition. In a clinically relevant in vivo model of prostate cancer composed of BPH1 cells and CAF, the application of HSP90 inhibitors reduced tumour growth, confirming and extending previous findings. Therefore we suggest that HSP90 is a potential target not only in tumour cells but also in CAF as part of the TME.

## 4. Materials and Methods

### 4.1. Tissue and Cells

PCa patients undergoing transurethral resection of the prostate (TURP) provided informed written consent for donating tissue, which was approved by the Eastern Multicentre Research Ethics Committee, MREC 02/5/63. The grading of the tumour is contemporary but based on the initial suggestion [54]. Clinical parameters and CAF experimental design are listed in Supplementary Table S1. CAF were grown as described previously and used for experiments at passage numbers 4 and 5, minimising epithelial contamination and senescence, consistent with previously reported studies [1]. BPH1 cells are initiated but non-tumourigenic cells, while WPMY-1 cells are immortalised human prostatic myofibroblasts [55,56].

### 4.2. Chemicals

Cell culture reagents were from Life Technologies (Paisley, UK), chemicals and small inhibitory compounds from Sigma-Aldrich (Poole, UK or St. Louis, MO, USA). The compound dipalmitoylradicicol was a generous gift, kindly provided by Dr. Akihiro Kitamura (Daiichi Sankyo, Tokyo, Japan). The collagen solution used for 3D-assays was made using a previously published protocol [57].

### 4.3. Cell Culture Assays

For 3D collagen gel contraction (CGC) assays, CAF were seeded into a collagen lattice at a final concentration of 25,000 cells/mL in 1 mg/mL final collagen concentration in 24 well plates, the suspension (0.5 mL) allowed to gel for 30–45 min at 37 °C, followed by addition of 1 mL medium per well (DMEM with 5% FCS). Gels were dislodged from the walls and bottom of the dish and photographed after 24 and 48 h of incubation with a Leica MZ6 stereo microscope and attached Leica ICA camera (Leica Microsystems, Deerfield, IL, USA). Experiments were performed in triplicate with 1–3 repetitions per CAF population.

Cell viability after CGC was checked with the CellTiter 96^®^ AQueous One Solution Cell Proliferation Assay (MTS) (Promega, Southampton, UK). The gels were washed 3× with PBS and then incubated with 300 µL medium and 60 µL reagent for two hours. 120 µL of the gently mixed medium/reagent mixture was transferred into a clean 96 well plate and the absorbance measured on a UV-vis spectrophotometer.

A scratch assay was performed on confluent CAF in 2D in 24 well plates by scratching with a pipette tip two crosses into the cell layer. After gentle washing with PBS, the cells were covered with 1 mL/well DMEM that contained only 1% FCS so as to avoid cell senescence due to serum starvation. Wounds were imaged at 0 and 24 hours with an Axiovert 200M inverted microscope, 5× plan-neofluar objective and an Axiocam MR3, and analyzed the images via Axiovision Software 4.8 (all Zeiss, Jena, Germany) and ImageJ [58]. Quadruplicate measurement per CAF population were performed and several different CAF populations were analysed.

### 4.4. Multiplex Assays

The methodology of bead-based multiplex assays is similar to ELISA using small beads, which in turn are analysed for their fluorescence via multi-channel flow cytometry. Here, cells were seeded into 24 well plates and the following day treatment was started in 1 mL medium (DMEM, 1% FCS) per well for 24 h. Supernatants were recovered, centrifuged for the removal of debris, and stored at −80 °C until use. The Cells were lysed with RIPA supplemented with protease and phosphatase inhibitors and also kept frozen at −80 °C until use. Aliquots of the lysates were quantified by using the DC Protein Assay kit (BioRad, Hertfordshire, UK) according to manufacturer’s instructions. We used the Milliplex Map Kit for measuring secretion of TGFβ1,2,3 (Millipore, Billercia, MA, USA) and the Widescreen BeadPlex Human Heat Shock Protein Panel (Merck, Darmstadt, Germany) on a BioPlex 200 System (BioRad, Hercules, CA, USA) according to manufacturers’ instructions.

### 4.5. In Vivo Studies

All animal experiments were approved by the Vanderbilt IACUC. Young male CB-17/IcrHsd-Prkdc-SCID mice, were purchased from Harlan (Dublin, VA, USA). Recombinant xenografts were made by mixing 1 × 10^5^ BPH1 cells and 2.5 × 10^5^ CAF per graft in collagen solution, allowed to gel, covered with medium and cultured overnight. The following day recombinants were grafted to the subrenal capsule site under isoflurane anaesthesia. Two grafts per kidney per animal were transplanted, as described previously [1]. For each CAF population and dose group, three grafts were transplanted. In addition, mice received a subcutaneous transplant that slowly released testosterone to raise the endogenous levels to those comparable in humans, as described previously [1]. Tumours were allowed to form over eight weeks, and then treated for four weeks with three different doses of dipalmitoyl-radicicol (50, 100 and 200 mg/kg) and 17-DMAG (5, 10 and 20 mg/kg) via intraperitoneal injections of compounds in sesame oil every four days. After 12 weeks in total, the mice were sacrificed, their kidneys resected, grafts cut in half and photographed before processing for histology. Graft dimensions were measured and the resultant tumour volume was calculated using the formula; volume = width × length × depth × π/6. This formula represented a conservative approach to evaluate tumour volumes, as it understates the volume of large, invasive tumours compared with smaller, non-invasive tumours. Resected grafts were fixed in 10% formalin, embedded in paraffin and processed for immunohistochemistry.

### 4.6. Immunohistochemistry and Staining of Tissue Sections

Human TURP samples and mouse kidneys with grafts attached were fixed in formaldehyde solution, processed and embedded in wax. Details of IHC were described previously [24]. Picrosirius red staining was performed as previously described [28]. The Ki67 evaluation was performed by examining three images per graft using the free application ImmunoRatio for automated image analysis [59].

### 4.7. Statistics

Statistical analysis performed with GraphPad Prism Software (San Diego, CA, USA) with tests as indicated in the text or figure legends. A *p* < 0.05 was considered statistically significant. *p*-levels are indicated by asterisks with *p* < 0.05 as *, *p* < 0.01 as ** and *p* < 0.001 as ***. Graphs show the mean plus the S.E.M.

## 5. Conclusions

Our studies support the hypothesis that HSP90 inhibitors may act upon CAF in addition to direct effects upon tumour epithelia. We demonstrate that HSP90 inhibitors can inhibit CAF contractility and motility in vitro, and that further investigation is warranted.

## Supplementary Materials

The following are available online at http://www.mdpi.com/2072-6694/8/9/77/s1. Figure S1: Histology of CAF/BPH1 tumours after treatments with HSP90 inhibitors in vivo, Figure S2: Contractility of CAF from patients of different age and Gleason grade, Figure S3: Reconstituted tumour sizes from CAF showing minimal and maximal contractility, Figure S4: Effects of Dorsomorphin upon CAF contractility, Figure S5: Effects of HSP90 inhibitors upon cellular levels of HSP27, Phospo-HSP27, and HSP60, Table S1: Collagen contractility and Gleason grade and patient age, Table S2: Tumour size elicited by most versus least contractile CAF.

## Abbreviations

The following abbreviations are used in this manuscript:

CAF	Cancer associated fibroblastCGC: Collagen gel contraction
GS	Gleason Score
HSP	Heat shock protein
PCa	Prostate cancer
TME	Tumour microenvironment
WC	Wound closure
