# Supplementary material for: Reduced Contractility and Motility of Prostatic Cancer-Associated Fibroblasts after Inhibition of Heat Shock Protein 90
Source: Cancers (Basel). 2016 Aug 24;8(9):77. doi: 10.3390/cancers8090077 (PMC5040979; doi:10.3390/cancers8090077)
Supplement: Supplementary file 1 [file cancers-08-00077-s001.pdf]

Supplementary Materials: Reduced Contractility and Motility of Prostatic Cancer-Associated Fibroblasts after Inhibition of Heat Shock Protein 90

Alexander Henke, Omar E. Franco, Grant D. Stewart, Antony C.P. Riddick, Elad Katz, Simon W. Hayward and Axel A. Thomson

Table S1. Collagen contractility and Gleason grade and patient age. Collagen contractility of CAF derived from patients of different Gleason score was examined, however there was no statistically significant difference in contractility when measured as a group. Similarly, there was no correlation between patient age and CAF contractility when compared as a group.

CAF population	AGE	Gleason Score	Test of tumour induction in vivo	Gel size in contraction assay (%)			Use of CAF lines in assays					
				0 hours	24 hours	48 hours	CGC radicicol	CGC 17-DMAG	CGC Y-27632	CGC Fasudil	Scratch assay	Bead assay
A	87	7	not tested	100	84.6	58.2						
B	64	9	yes	100	67.8	49.2						
C	82	6	not tested	100	77.8	66.7		yes	yes	yes		
D	83	6	yes	100	73.9	53.0	yes				yes	yes
E	74	8	yes	100	90.1	77.7		yes	yes	yes		
F	62	poorly differentiated	yes	100	76.1	68.3	yes				yes	yes
G	84	poorly differentiated	yes	100	58.5	46.6	yes					
H	74	7	not tested	100	61.3	47.5	yes					
I	83	9	not tested	100	74.8	64.7	yes					
J	81	10	not tested	100	76.6	64.3	yes					
K	85	7	yes	100	54.1	43.1						
L	87	7	not tested	100	73.3	63.7						
M	72	7	yes	100	60.5	46.6	yes					
N	53	9	yes	100	84.2	68.0	yes	yes	yes	yes	yes	yes
O	69	9	not tested	100	76.9	70.6						
P	70	10	not tested	100	79.4	70.4						
Q	84	9	not tested	100	74.5	57.1						
R	70	8	yes	100	51.9	43.7	yes					
S	72	7	not tested	100	75.9	63.3	yes					
T	83	8	yes	100	68.7	55.5	yes				yes	yes

Table S2. Tumour size elicited by most versus least contractile CAF. The three most and least contractile CAF lines were tested in tumour reconstitution with BPH1 cells to examine whether contractility was associated with tumour size in vivo. There appeared to be a trend towards larger tumour size with most contractile CAF, however this was not statistically significant. Each CAF line was grafted in triplicate and all single values are shown. Due to high intra-group variance, the difference did not reach the level of significance ($p = 0.15$).

	**Tumour volumes in current study after 3 months (mL)**							**Historic tumour weights (Olumi et al. 1999) (g)**			
	**Controls**	**Radi 50**	**Radi 100**	**Radi 200**	**DMAG 5**	**DMAG 10**	**DMAG 20**	**BPH1 only**	**CAF+BPH1**	**CAF only**	**NPF+BPH1**
Graft 1	0.065	0.020	0.016	0.025	0.120	0.019	0.067				
Graft 2	0.063	0.029	0.024	0.041	0.151	0.023	0.032				
Graft 3	0.090	0.087	0.084	0.036	0.014	0.031	0.068				
Graft 4	0.393	0.111	0.017	0.034	0.053	0.014	0.024				
Graft 5	0.661	0.047	0.085		0.070	0.010	0.032				
Graft 6	0.115	0.139	0.066		0.116	0.026	0.023				
Graft 7			0.006				0.009				
**n**	**6**	**6**	**7**	**4**	**6**	**6**	**7**	**6**	**9**	**8**	**9**
median	0.102	0.067	0.024	0.035	0.110	0.021	0.032	0.016	0.375	0.004	0.007
mean	0.231	0.072	0.042	0.034	0.102	0.021	0.037				
SD	0.245	0.048	0.034	0.006	0.036	0.008	0.022				
SEM	0.100	0.020	0.013	0.003	0.015	0.003	0.008				
min	0.063	0.020	0.006	0.025	0.053	0.010	0.009	0.004	0.175	0.002	0.003
max	0.661	0.139	0.085	0.041	0.151	0.031	0.068	0.027	1.250	0.005	0.012

Excluded from evaluation: outlier tumour of V = 4.4 mL in the control group of the current study.

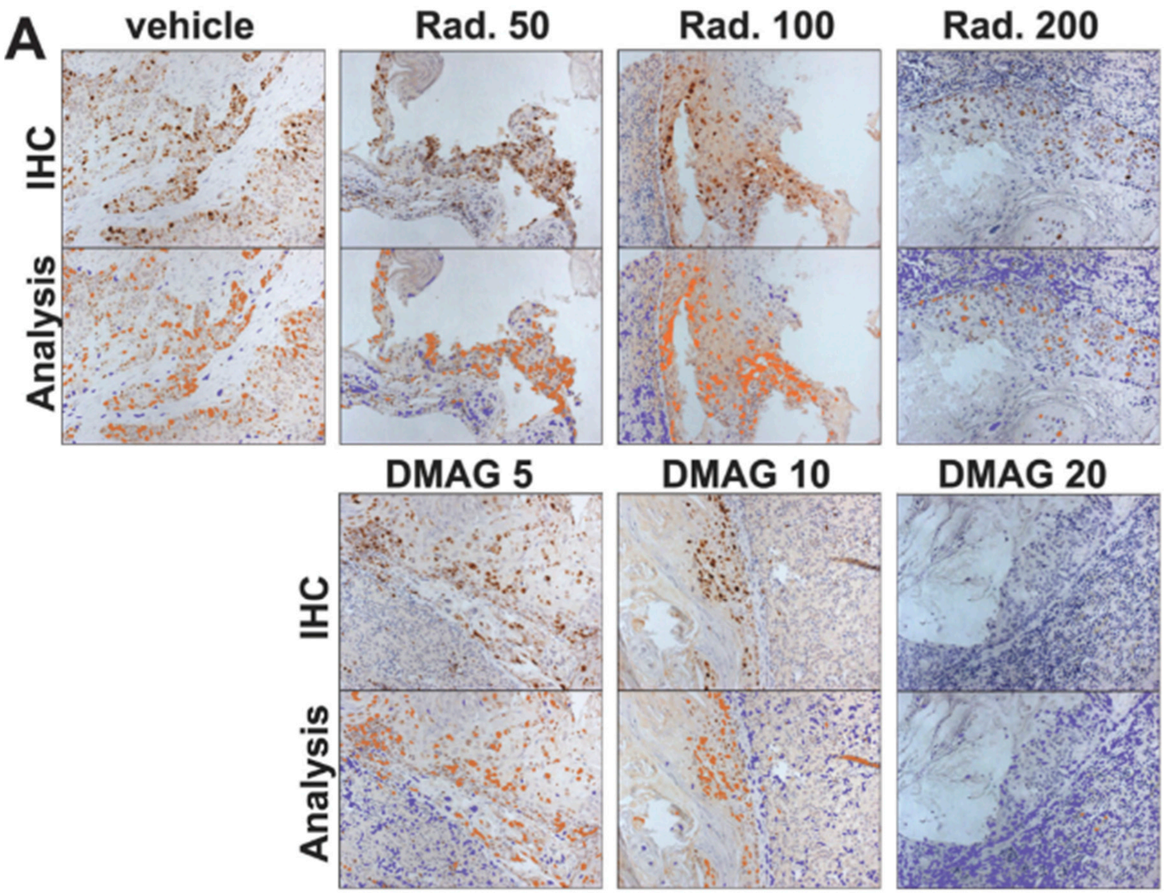

Figure S1. Histology of CAF/BPH1 tumours after treatments with HSP90 inhibitors in vivo. To examine effects upon cell proliferation, tissue sections of CAF/BPH1 tumours were stained for the proliferation marker Ki67 (labelled IHC). Images were analysed via software to quantify Ki67 expression in nuclei by pseudocoloring positive nuclei followed by counting (labelled Analysis). Ki67-positive nuclei were stained dark brown for IHC and presented in false colour beige in the analysis images. The top rows shows IHC for tumours treated with dipalmitoyl-radicicol, while the lower row shows tumours treated with 17-DMAG.

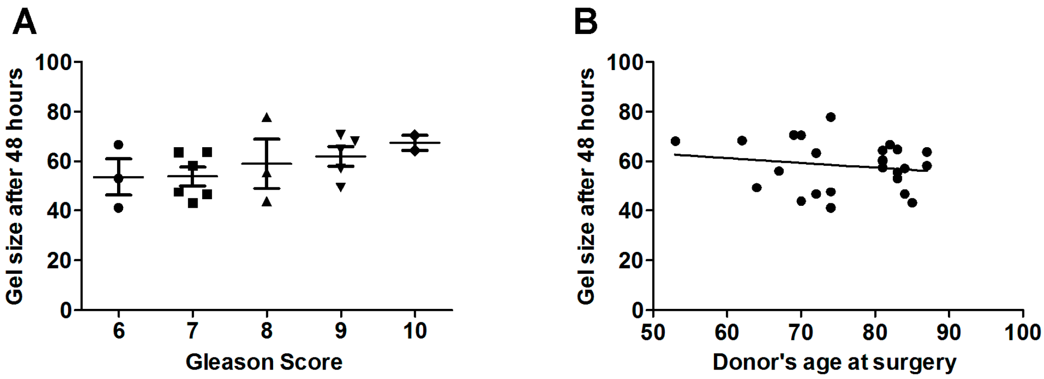

Figure S2. Contractility of CAF from patients of different age and Gleason grade. (**A**) shows gel size and contractility of CAF isolated from patients of Gleason score 6, 7, 8, 9 and 10, there were no statistically significant differences among the groups; (**B**) shows gel size and contractility of CAF isolated from patients of increasing age.

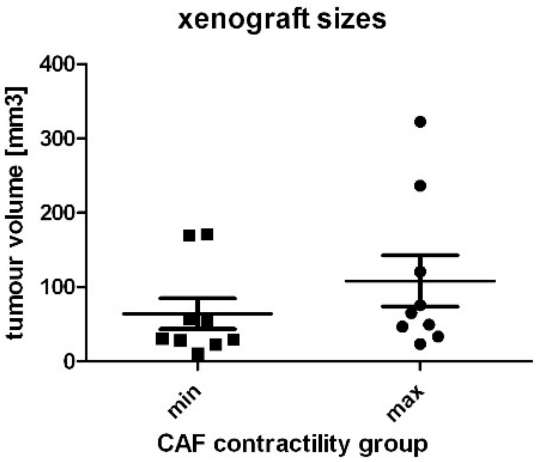

Figure S3. Reconstituted tumour sizes from CAF showing minimal and maximal contractility. CAF were combined with BPH1 cells and grafted sub-renally to compare tumour size between the least and most contractile CAF.

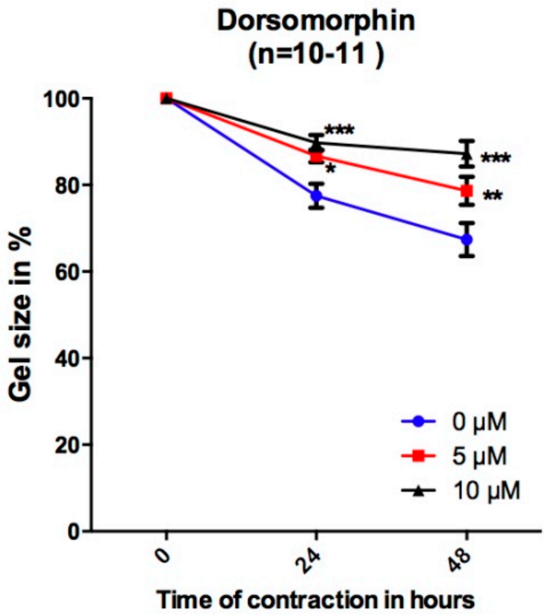

Figure S4. Effects of Dorsomorphin upon CAF contractility. CAF contractility was assessed in the presence of 0, 5 or 10µM Dorsomorphin, which showed a significant inhibition of CAF contractility. Dorsomorphin is in inhibitor of BMP signaling, however, other BMP inhibitors had no effect upon CAF contractility, and we speculate that it was an off target effect of dorsomorphin upon HSP90 which led to the effects upon contractility. Asterisks denote the level of significance: * for $p < 0.05$, ** for $p < 0.01$ and *** for $p < 0.001$.

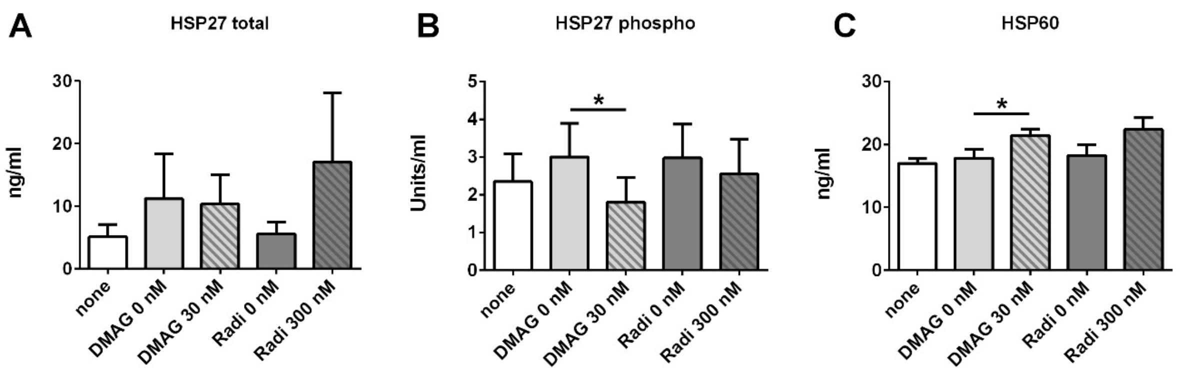

Figure S5. Effects of HSP90 inhibitors upon cellular levels of HSP27, Phospo-HSP27, and HSP60. We examined intracellular protein levels of heat shock proteins in CAF after treatment with HSP90 inhibitors. Lysates of treated CAF were subjected to a multiplex bead assay for HSP27, phospo-HSP27 and HSP60. Treatment with 30nM 17-DMAG led to a reduction in phospo-HSP27 and an increase in HSP60.

© 2016 by the authors. Submitted for possible open access publication under the terms and conditions of the Creative Commons Attribution (CC-BY) license (http://creativecommons.org/licenses/by/4.0/).
